# Three Cases of Rattlesnake Bites

**Published:** 1890-01

**Authors:** 


THREE CASES OF RATTLESNAKE BITES.—Dr. E. Duges, gives three cases of rattlesnake bites. Treatment: injection of four grammes (ʒi) of a concentrated solution of carbolic acid; internally, jarborandi and infusion of coca; massage with camphorated oil; next day, ligature removed; good food. When the wound, which was on the left hand, was inflicted, the man, who was seventy years of age, immediately ligated his left arm above the wrist and went to the doctor. Considerable œdema of the hand ensued. Superficial incisions were made, with profuse bleeding. Gangrene of the skin on the back of the hand followed. Recovered in one month.

Second case, man, sixty years old; bite over right ankle; immediate ligature; same injection; advised to get drunk, which he gladly did. Next morning all right.

Third case, man, thirty-seven years old; bite on right little finger; ligature; same injection twenty-four hours after accident; plenty of brandy. Next day much better; much œdema; incisions; poultices of *Datura stramonium*, and mercurial ointment. Recovered on the eighth day. The considerable quantities of carbolic acid produced no constitutional symptoms; the blood was quite thin.—*Boletin de Medicina*, Guanajuato.

